# A study of cancer risks in relatives of patients with serrated polyposis

**DOI:** 10.1186/1897-4287-10-S2-A21

**Published:** 2012-04-12

**Authors:** AK Win, RJ Walters, DD Buchanan, MA Jenkins, K Sweet, DM McKeone, MD Walsh, M Clendenning, SA Pearson, E Pavluk, B Nagler, JL Hopper, N Walker, C Rosty, S Parry, JP Young

**Affiliations:** 1 Centre for MEGA Epidemiology, School of Population Health, University of Melbourne, Carlton, VIC 3053, Australia; 2 Familial Cancer Laboratory, Queensland Institute of Medical Research, Herston QLD 4006, Australia; 3 School of Medicine, University of Queensland, Herston QLD 4006, Australia; 4 Division of Human Genetics, Department of Internal Medicine, Comprehensive Cancer Center, The Ohio State University, Columbus, OH 43221, USA; 5 Envoi Pathology, Herston, Q4006, Australia; 6 Department of Molecular and Cellular Pathology, University of Queensland, Herston, Q4006, Australia; 7 Department of Gastroenterology, Middlemore Hospital, Auckland, New Zealand; 8 New Zealand Familial Gastrointestinal Cancer Registry, Auckland Hospital, New Zealand

## Objectives

Serrated polyposis (hyperplastic polyposis) is characterized by multiple polyps with serrated architecture in the colorectum. While patients with serrated polyposis are known to be at increased risk of colorectal cancer (CRC) and possibly extracolonic cancers, cancer risks for their relatives have not been widely explored. The aim of this study was to estimate the risks of CRC and extracolonic cancers for relatives of patients with serrated polyposis.

## Methods

A cohort of the 1,639 first- and second-degree relatives of 100 index patients with serrated polyposis recruited regardless of a family history of polyps or cancer from genetic clinics in Australia, New Zealand, Canada and the USA, were retrospectively analysed to estimate the country-, age- and sex-specific standardized incidence ratios (SIRs) for relatives compared with the general population.

## Results

A total of 102 CRCs were observed in first- and second-relatives (SIR 2.25, 95% confidence interval, CI 1.75–2.93; *P*<0.001), with 54 in first-degree relatives (SIR 5.16, 95% CI 3.70–7.30; *P*<0.001) and 48 in second-degree relatives (SIR 1.38, 95% CI 1.01–1.91; *P*=0.04). The SIR of CRC for relatives was greater when index cases were diagnosed under age 50 (*P*=0.04). Six pancreatic cancers were observed in first-degree relatives (SIR 3.64, 95% CI 1.70–9.21; *P*=0.003). There was no evidence of increased risk for cancer of the stomach, brain, breast or prostate.

## Conclusions

Our finding that relatives of serrated polyposis patients are at significantly increased risk of colorectal and pancreatic cancer, adds to the accumulating evidence that serrated polyposis has an inherited component.

